# Supplementary figures and images for: Functionally heterogeneous human satellite cells identified by single cell RNA sequencing
Source: eLife. 2020 Apr 1;9:e51576. doi: 10.7554/eLife.51576 (PMC7164960; doi:10.7554/eLife.51576)

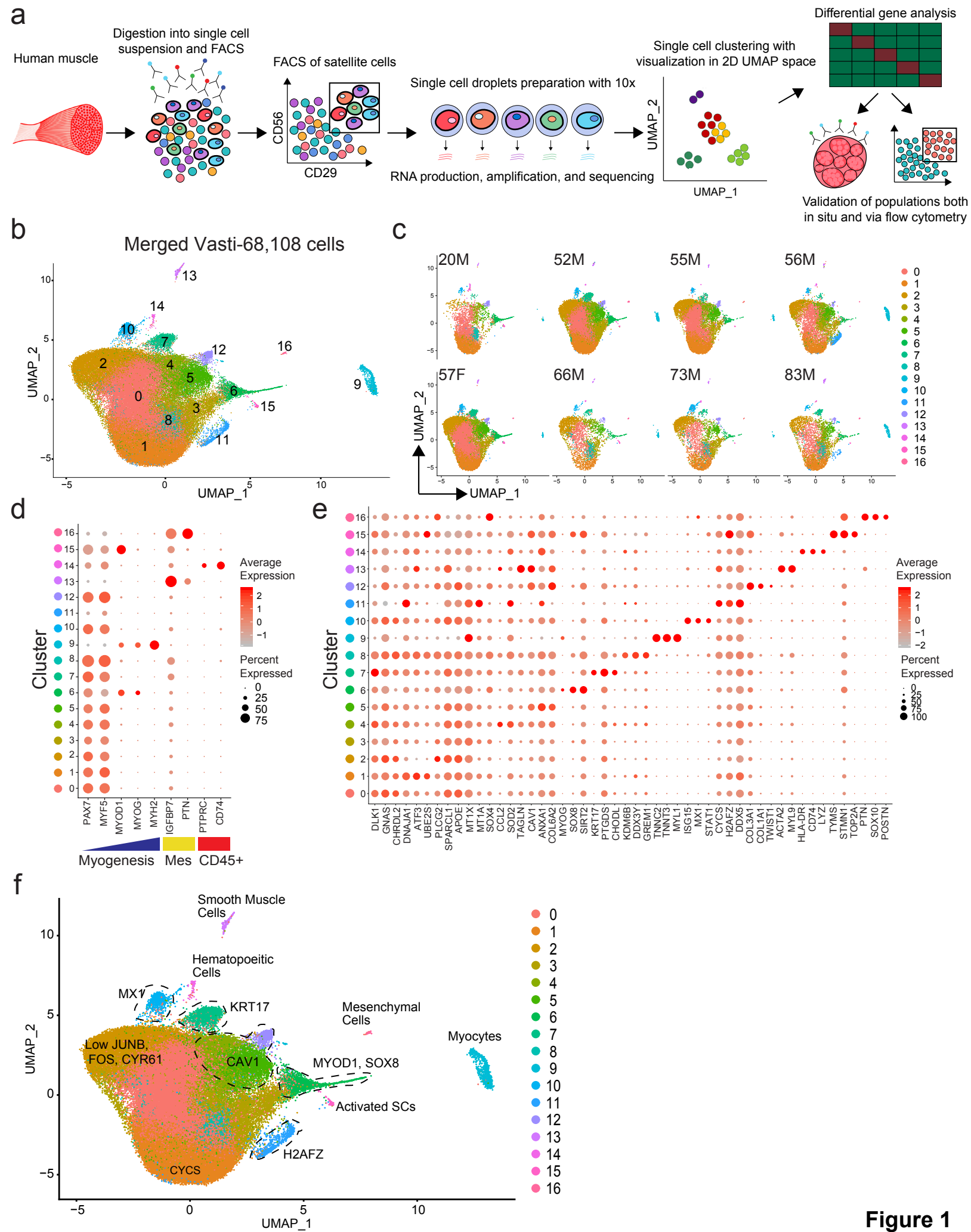

**Figure 1**

Supplement: Figure 1—source data 1. [file elife-51576-fig1-data1.pdf]

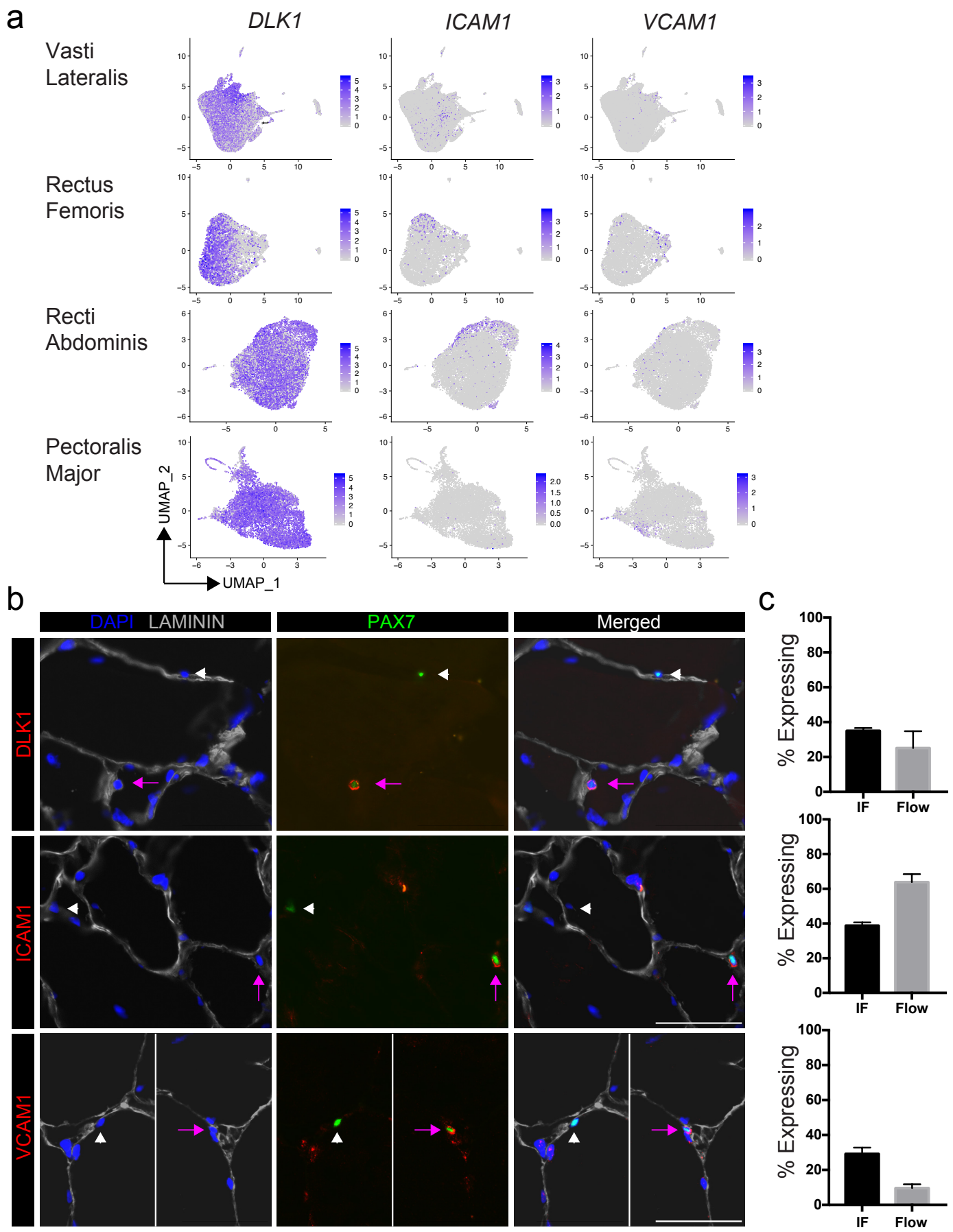

Figure 2

Supplement: Figure 2—source data 1. [file elife-51576-fig2-data1.pdf]

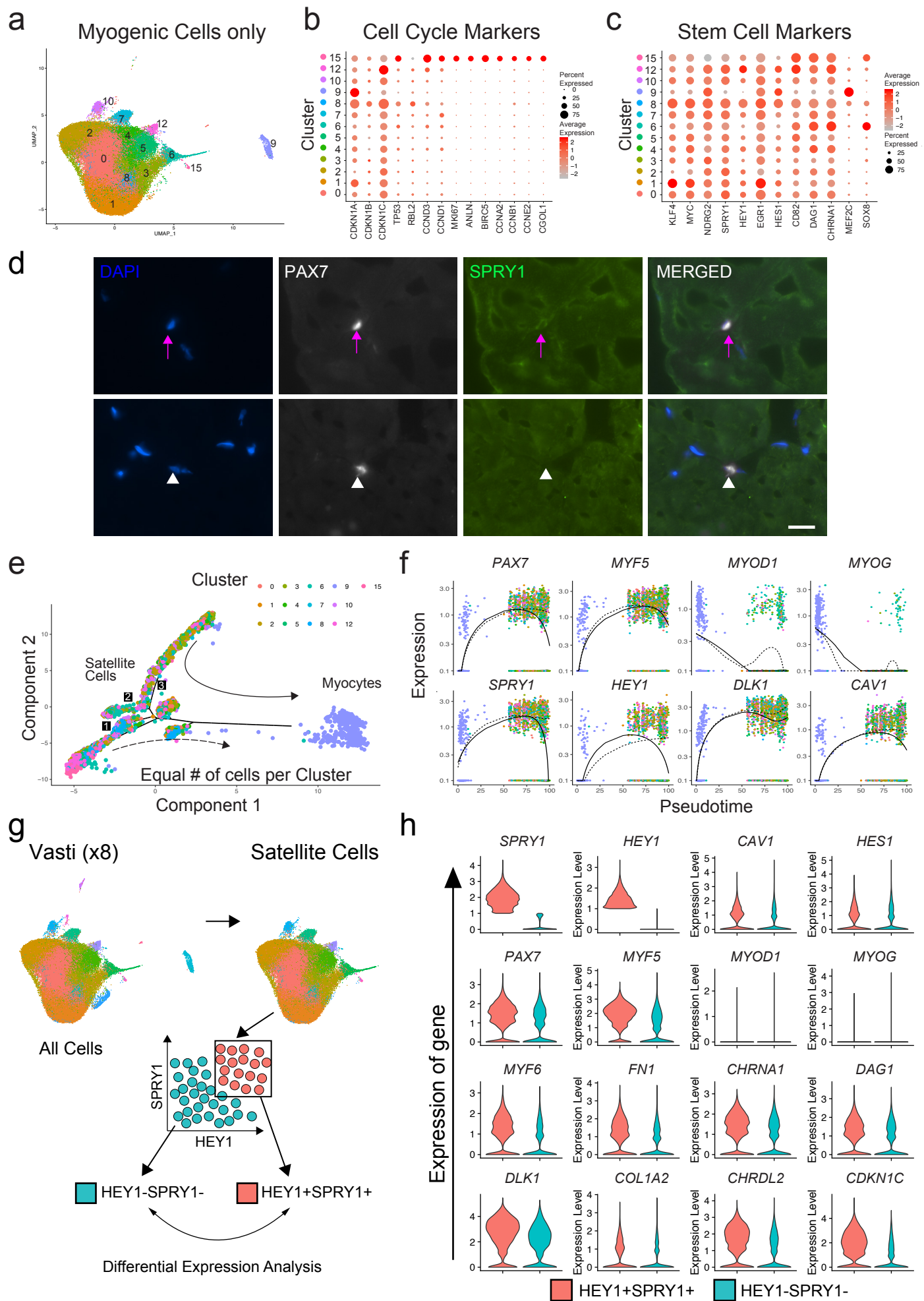

**Figure 3**

Supplement: Figure 3—source data 1. [file elife-51576-fig3-data1.pdf]

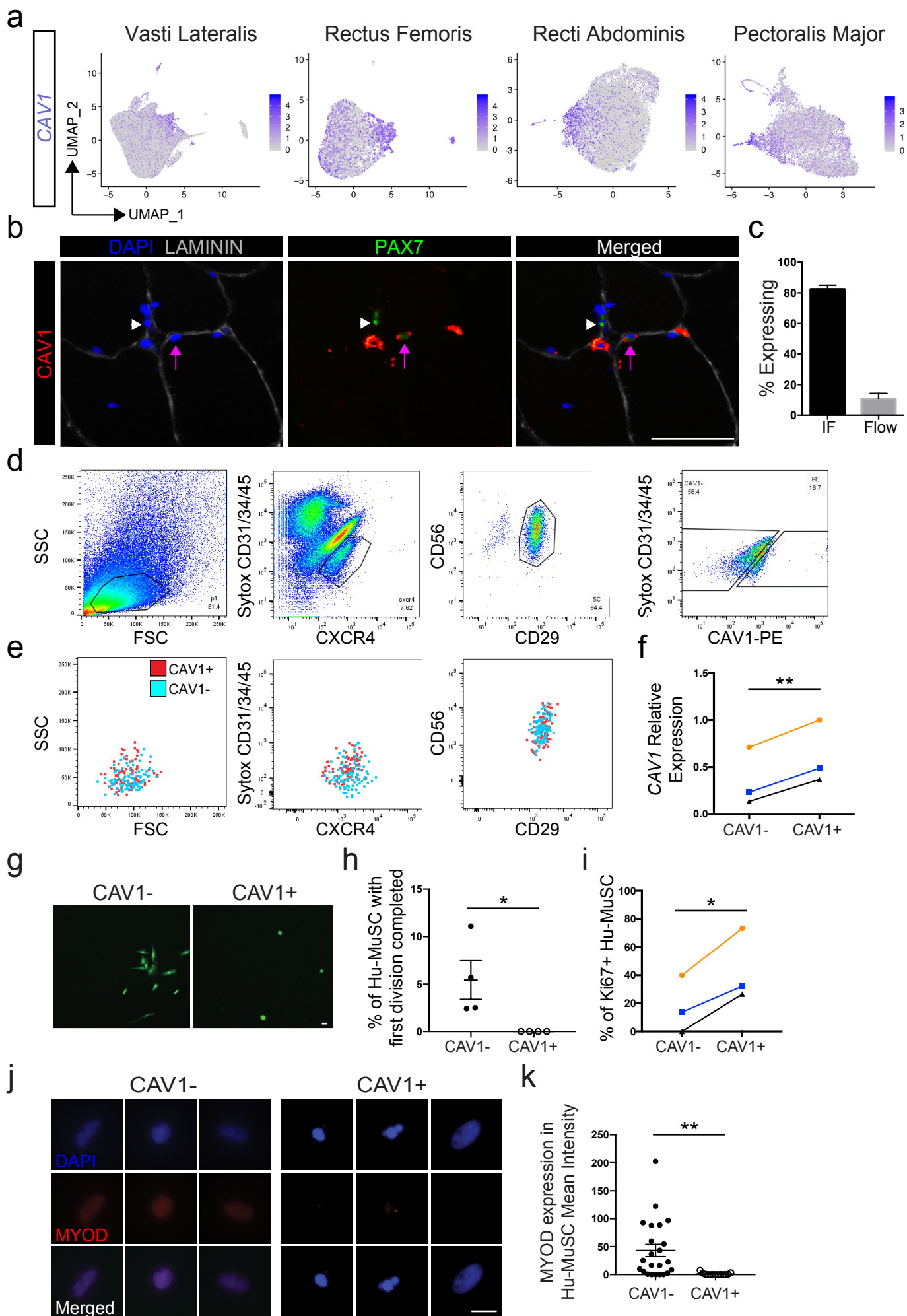

**Figure 5**

Supplement: Figure 5—source data 1. [file elife-51576-fig5-data1.pdf]

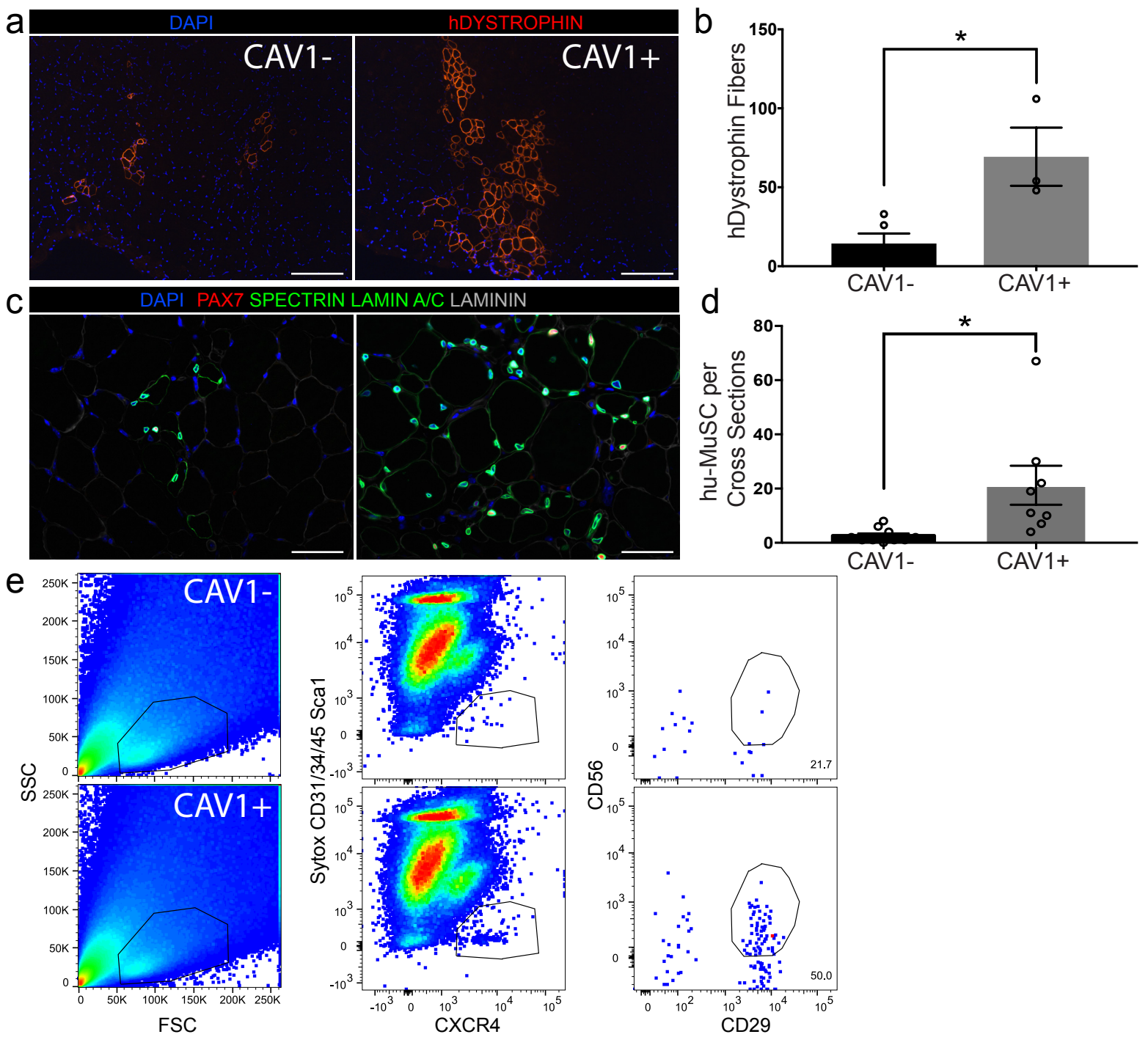

Figure 6

Supplement: Figure 6—source data 1. [file elife-51576-fig6-data1.pdf]
